# The Sugar Transporter Gene Family in Colored Calla Lily: Identification, Expression Patterns, and Roles in Soft Rot Disease

**DOI:** 10.3390/plants14172631

**Published:** 2025-08-24

**Authors:** Xiaorong Huang, Zhen Zeng, Yushan Lu, Yi Wang, Menghan Zhang, Lele Wu, Wei Tian, Defeng Chen, Guojun Zhang, Zunzheng Wei

**Affiliations:** 1 College of Horticultural Science & Technology, Hebei Normal University of Science & Technology, Qinhuangdao 066004, China; huangxiaorongbaafs@163.com (X.H.);; 2 Institute of Grassland, Flowers and Ecology, Beijing Academy of Agriculture and Forestry Sciences, Beijing 100097, China; zengzh2020@163.com (Z.Z.);

**Keywords:** STP, gene family, colored calla lily, soft rot

## Abstract

Carbohydrates are a primary nutrient for plant growth, and sugar transporter proteins play a crucial role in sugar allocation. In this study, hexose transporter genes encoding in the genome of colored calla lily ‘Jingcai Yangguang’ (*Zantedeschia elliottiana* cv. Jingcai Yangguang) were identified, and their expression patterns following infection by *Pectobacterium carotovora* subsp. *Carotovora* were investigated. Additionally, the transport characteristics of three hexose transporters, ZeSTP7, ZeSTP15, and ZeSTP17, were determined. The results showed that the sugar transporter protein family in *Z. elliottiana* comprises 18 members, most of which possess 12 transmembrane domains. Phylogenetic analysis revealed that the *ZeSTP* gene family was divided into five subgroups. Tandem gene duplication events were identified on the 16 chromosomes of *Z. elliottiana*, with multiple tandemly duplicated genes detected. Comparative analysis of synteny between species identified *ZeSTP8* and *OsSTP22* as homologous gene pairs, while *OsSTP6* (*OsMST6*) was identified as a homologous gene pair with both *ZeSTP14* and *ZeSTP17*. Following infection by *P. carotovora* subsp. *carotovora*, the transcript levels of *ZeSTP7*, *ZeSTP15*, and *ZeST17* were all significantly elevated. Yeast mutant hexose complementation tests indicated that ZeSTP7 could transport glucose and galactose, whereas ZeSTP15 and ZeSTP17 exhibited limited transport capacity in this respect. This study provides a systematic identification and analysis of hexose transporter genes at the genome-wide level, highlighting the role of *ZeSTP* genes in the response of colored calla lily to soft rot and laying a theoretical foundation for further understanding the functions of sugar transporter genes.

## 1. Introduction

Sugars are primary nutrients and products of photosynthesis in plants, primarily stored as sucrose and transported through the phloem to various plant tissues, facilitating sugar translocation from source to sink organs [1,2]. The intercellular transmembrane transport of sugars relies on specific carriers known as sugar transport proteins [3]. Currently, three main types of sugar transport proteins have been identified in plants: monosaccharide transporters (MSTs), sucrose transporters (SUC/SUTs), and sugar will eventually be exported transporters (SWEETs) [4]. Both SUTs and MSTs belong to the major facilitator superfamily (MFS) [5].

In most plants, sucrose synthesized in mesophyll cells through photosynthesis is transported from the source to sink organs. A portion of the sucrose is stored, while the rest is hydrolyzed back into monosaccharides and further absorbed by sink organs with the assistance of monosaccharide transporters [6]. Sugar transport protein/hexose transporters (STPs), as the primary members of the MST family, typically possess 12 α-helices forming transmembrane domains and function as H+/sugar cotransporters, primarily responsible for transporting hexoses from the apoplast into cells [7]. Additionally, hexose transporters are involved in plant growth and development, pollen germination, and the transport of sugars during biotic and abiotic stress responses in plants.

*STPs* are important regulators of plant growth and development. In Arabidopsis, the expression level of *AtSTP1* is high during seed germination (particularly during seed imbibition) and in the early stages of seedling growth (1–7 days after germination). After germination, *AtSTP1* is highly active in the roots and may play a crucial role in seedling growth by regulating the uptake of monosaccharides in the roots [8]. *AtSTP1* exhibits circadian expression in guard cells; it mediates the uptake of glucose and other monosaccharides via osmotic adjustment to modulate stomatal movement and reallocate carbon skeletons [9,10]. *AtSTP13* and *AtSTP14* are subject to similar circadian control, being strongly induced at night and repressed under continuous light [7]. *AtSTP7* supplies pentoses and arabinose to the cell wall [11]; *AtSTP13* participates in programmed cell death [12]. Thus, *STPs* are essential for plant growth. Furthermore, during pollen and pollen-tube development, *AtSTP9* (early phase) [13] and *AtSTP6* (late phase) [14] act sequentially to supply a sustained carbon flux to the pollen grain and elongating tube, and *AtSTP4* continuously imports apoplastic monosaccharides into sink cells of the root apex and anther, ensuring a steady carbon supply [15]. In fruits and storage organs, *VvHT1* drives hexose import in young grape berries, thereby setting the final sugar-to-acid ratio [16], and fifteen *MeSTPs* in cassava coordinately promote early tuberous expansion [17]. Under environmental stress, *STPs* contribute to plant defense: *AtSTP3* and *AtSTP4* transcripts are rapidly and transiently elevated after mechanical wounding [18], and *OsSTP14* is strongly upregulated in rice subjected to flooding and high-temperature stress [19].

In higher plants, sugars are the main nutrients that pathogens extract from host plants. Therefore, competition for sugar allocation occurs between plants and pathogens, with sugar transport proteins playing a key role in this process [20]. Several hexose transporters have been confirmed to participate in sugar transport during plant–pathogen interactions. For example, in *Arabidopsis thaliana*, *AtSTP4* is expressed in mature leaves infected by fungi [21]. *AtSTP13* is induced by *Botrytis cinerea* and actively absorbs hexoses to support host plant defense against gray mold invasion [12]. The phosphorylation-dependent regulation of *AtSTP13* is crucial for *A. thaliana* defense, and the double mutant *Atstp13* and *Atstp1* weakens *A. thaliana* resistance to *Pseudomonas syringae* [22]. In maize, *ZmSTP2* and *ZmSTP20* respond to *Fusarium graminearum*, and the double mutant *zmstp2* and *zmstp20* enhances maize susceptibility [23]. However, *STPs* play a negative regulatory role in resistance to powdery mildew in *A. thaliana* and stripe rust in wheat. For example, *AtSTP8* is localized on the endoplasmic reticulum in *A. thaliana* and is recruited to the extrahaustorial membrane during powdery mildew infection, thereby enhancing powdery mildew colonization on *A. thaliana* [24]. In wheat, *TaSTP6* is induced by abscisic acid, and overexpression of *TaSTP6* and *TaSTP13* promotes host plant susceptibility to wheat stripe rust and powdery mildew [25,26]. In summary, hexose transporters are involved in plant immune processes and have different roles in different plants.

Previous studies have reported genome-wide identification of the *STP* gene family in *A. thaliana*, rice (*Oryza sativa*), wheat (*Triticum aestivum*), and maize (*Zea mays* L.). The earliest identification of 14 *STP* genes was in *A. thaliana* [7], with most *AtSTPs* exhibiting broad substrate specificity. In rice, 28 *OsSTP* genes were identified [19]; 81 *TaSTP* genes were identified in wheat [27]; and 24 *ZmSTP* genes were identified in maize [23].

Calla lily (*Zantedeschia* spp.) is a perennial herbaceous flower belonging to Araceae, of which colored calla lily holds significant economic value [28]. However, bacterial soft rot caused by *Pectobacterium carotovora* subsp. *carotovora* significantly reduces the quality of colored calla lily [29,30]. Currently, there is no cure for calla lily soft rot, and its impact is primarily controlled through cultivation management, physical, and chemical means [31]. *STP* genes have been confirmed to play important roles in the interactions between multiple plants and pathogens. Despite in-depth research on the *STP* gene family in *A. thaliana* and wheat, a bioinformatics analysis of the *ZeSTP* gene family in colored calla lily is still lacking. Therefore, this study identified and analyzed the *ZeSTP* gene family in *Z. elliottiana* cv. Jingcai Yangguang, revealing the responsiveness and expression patterns of *ZeSTP* genes to *P*. *carotovora* subsp. *carotovora*. This work lays a theoretical foundation for further exploring the potential role of the *STP* family in the molecular breeding of colored calla lilies.

## 2. Results

### 2.1. Identification of ZeSTP Gene Family Members in Z. elliottiana

A total of 18 *ZeSTP* genes were identified through the analysis of the annotation information from the genome sequencing results of *Z. elliottiana*. These genes were sequentially named *ZeSTP1* to *ZeSTP18* based on their chromosomal locations in the genome. The molecular weights and isoelectric points of the STP family proteins in *Z. elliottiana* were analyzed. The results showed that the molecular weight ranged from 9.1 kDa (ZeSTP6) to 91.9 kDa (ZeSTP13), and the isoelectric points ranged from 5.1 (ZeSTP4) to 10.86 (ZeSTP1). Among them, ZeSTP2, ZeSTP4, and ZeSTP6 were acidic proteins, while the others were basic proteins. In addition, the transmembrane domains of the ZeSTP protein family were predicted, revealing that most proteins possess 12 transmembrane domains (TMD) (Table 1).

### 2.2. Phylogenetic Analysis of the ZeSTP Family in Z. elliottiana 

The differences in *STP* genes among species may arise from the expansion of gene families in different species. To explore this, a multiple sequence alignment of ZeSTP family protein sequences with *O. sativa*, *A. thaliana* was performed, and the phylogenetic tree was constructed.

Based on the branches of the phylogenetic tree, the *ZeSTP* family can be divided into five subgroups: group I, group II, group III, group IV, and group V. Each subgroup contains members from multiple species, indicating that the *STP* genes in these subgroups likely share a common evolutionary ancestor. The root node of the *STP* genes splits into two major branches, one leading to the ancestral gene of group V and the other to the common ancestral gene of groups I, II, III, and IV.

**Figure 1:**
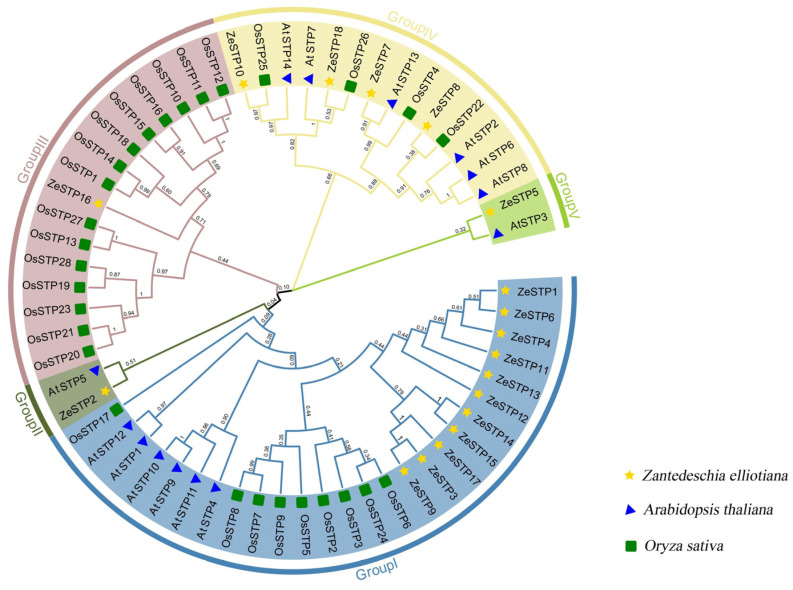
Phylogenetic tree of STP proteins in *O. sativa*, *A. thaliana*, and *Z. elliottiana*. Different-colored branches represent different groupings, while different-shaped patterns represent different species.

Gene expansion events may have occurred in *Z. elliottiana*. For example, in group I, the evolutionary branch of *ZeSTP12* further diverged into five *ZeSTP* genes (Figure 1). Additionally, in groups II and V, the *ZeSTPs* of *Z. elliottiana* are more closely related to the *AtSTPs* of *A. thaliana*, while in group III, the *ZeSTPs* of *Z. elliottiana* are more closely related to the *OsSTPs* of rice. In contrast, the distribution of *STP* members from *O. sativa*, *A. thaliana*, and *Z. elliottiana* is relatively even in group IV.

### 2.3. Analysis of Sequence Structural Characteristics of the ZeSTP Gene Family in Z. elliottiana

**Figure 2:**
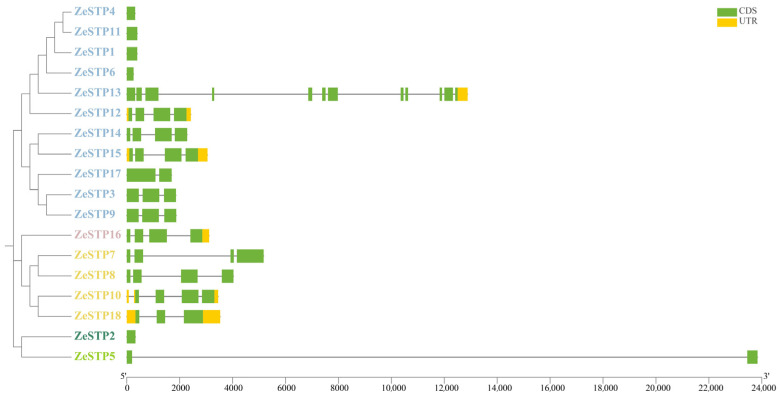
Gene structure of the *ZeSTP* family in *Z. elliottiana*. The green boxes, yellow boxes, and black lines represent coding sequences, non-coding regions, and gene lengths, respectively. Different-colored branches represent different groupings within the phylogenetic tree of the *ZeSTP* gene family.

Gene structure is crucial for the study of protein function. Therefore, in this study, the visual analysis of the sequence structure of the *ZeSTP* gene family in *Z. elliottiana* was performed. The results showed significant differences in the number of exons among the 18 *ZeSTP* genes (Figure 2). Among them, *ZeSTP13* has the highest number of exons with 12, while some *ZeSTP* genes (such as *ZeSTP4* and *ZeSTP11*) contain only one exon. The multiple exons in *ZeSTP13* may be the reason for its increased gene length. Additionally, an unusually long intron, exceeding 22 kb in length, was inserted into *ZeSTP5*, which is also a cause for its increased gene length. The length of most *ZeSTP* genes is within 6 kb. Among the 18 *ZeSTP* genes, some genes contain untranslated regions (UTRs) at both the 5′ and 3′ ends, such as *ZeSTP10*, *ZeSTP12*, *ZeSTP15*, and *ZeSTP18*; while some genes (such as *ZeSTP13* and *ZeSTP16*) have only one UTR region at the 3′ end, and most genes do not contain UTR regions (Figure 2).

**Figure 3:**
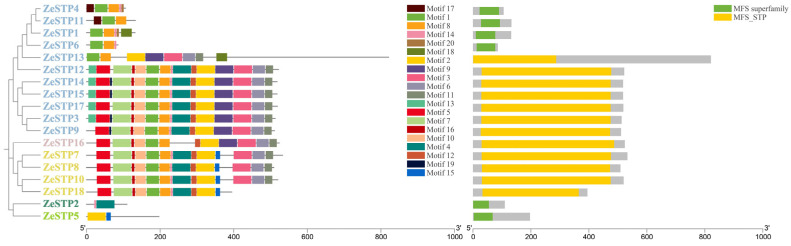
Distribution of conserved motifs and domains in the ZeSTP family of *Z. elliottiana*.

Conserved motif analysis revealed differences in conserved motifs among different evolutionary branches of the ZeSTP gene family. In the phylogenetic tree, ZeSTP genes within the same subgroup show similar conserved motifs, indicating sequence variation within conserved regions (Figure 3). However, even if the conserved motifs of a gene are low, the structural conservation of the protein may still be high. The conserved domains of the gene family can be divided into two categories: MFS superfamily and MFS_STP. For example, genes containing the MFS superfamily conserved domain, such as *ZeSTP4*, *ZeSTP11*, *ZeSTP1*, *ZeSTP6*, *ZeSTP2*, and *ZeSTP5*, have only one conserved domain in their coding sequence (CDS) region (Figure 2), and both the length of the conserved motifs and domains are less than 200 bp. In contrast, genes containing the MFS_STP conserved domain have more than one conserved domain in their CDS region (Figure 2), and both the length of the conserved motifs and domains are greater than 200 bp (Figure 3).

### 2.4. Chromosomal Localization Analysis of the ZeSTP Gene Family in Z. elliottiana

**Figure 4:**
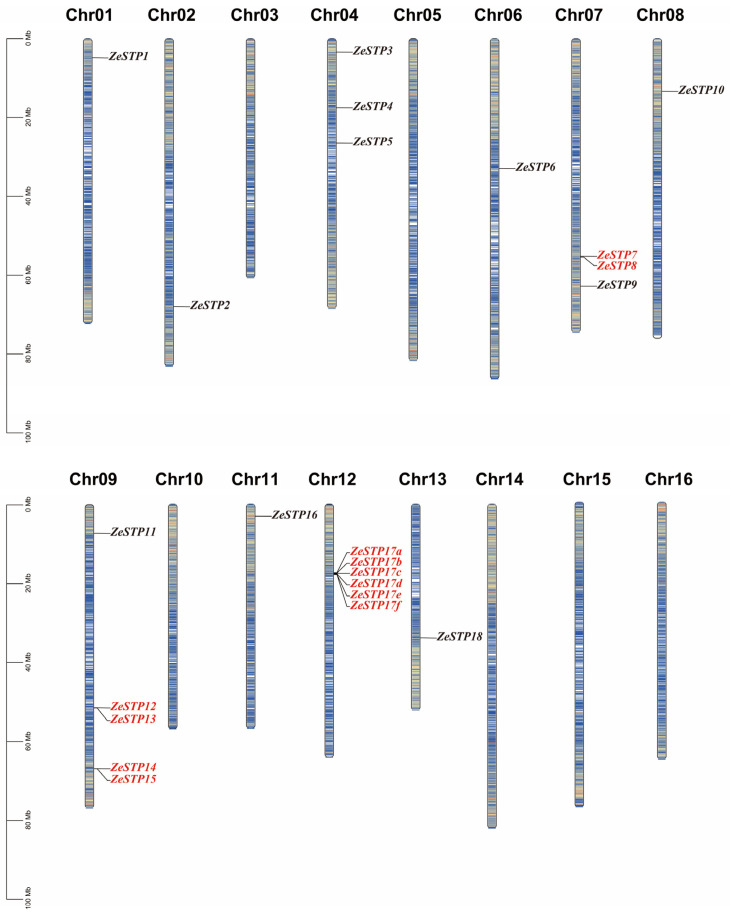
Chromosomal distribution of *STP* family genes in *Z. elliottiana*. The 18 *STP* family members of colored calla lily are labeled. The lines on the chromosomes represent gene density in the calla lily genome. Red lines indicate higher gene density, while blue lines indicate lower gene density.

To investigate the replication of the *STP* gene family in the genome of *Z. elliottiana*, a chromosomal distribution map of the *STP* gene family was created based on the chromosomal positions of the genes provided in the annotation file. The results showed that the distribution of *ZeSTP* genes across the 16 chromosomes is not uniform (Figure 4). *ZeSTP* genes are mainly concentrated on chromosomes 1, 2, 3, 4, 6, 7, 8, 9, 11, 12, and 13 (Figure 4), while the remaining six chromosomes do not contain any *ZeSTP* genes. Additionally, *ZeSTP* genes on chromosomes 1, 4, 6, 8, 11, and 12 are predominantly located at the upper ends of the chromosomes, whereas those on chromosomes 2, 7, 9, and 13 are mostly found at the lower ends.

It is shown that *ZeSTP7* and *ZeSTP8* on chromosome 7 may have undergone tandem gene duplication events. Similarly, chromosome 9 contains five *ZeSTP* genes, among which *ZeSTP12* and *ZeSTP13* have experienced tandem duplication events, as well as *ZeSTP14* and *ZeSTP15*. Furthermore, the *ZeSTP17* gene on chromosome 12 may have undergone multiple tandem duplication events, resulting in six *ZeSTP17* genes encoding the same CDS region (Figure 4). Tandemly duplicated genes play an important role in the evolutionary process of species.

### 2.5. Intraspecific Collinearity Analysis of STP Genes in Z. elliottiana and Interspecific Collinearity Analysis with O. sativa

**Figure 5:**
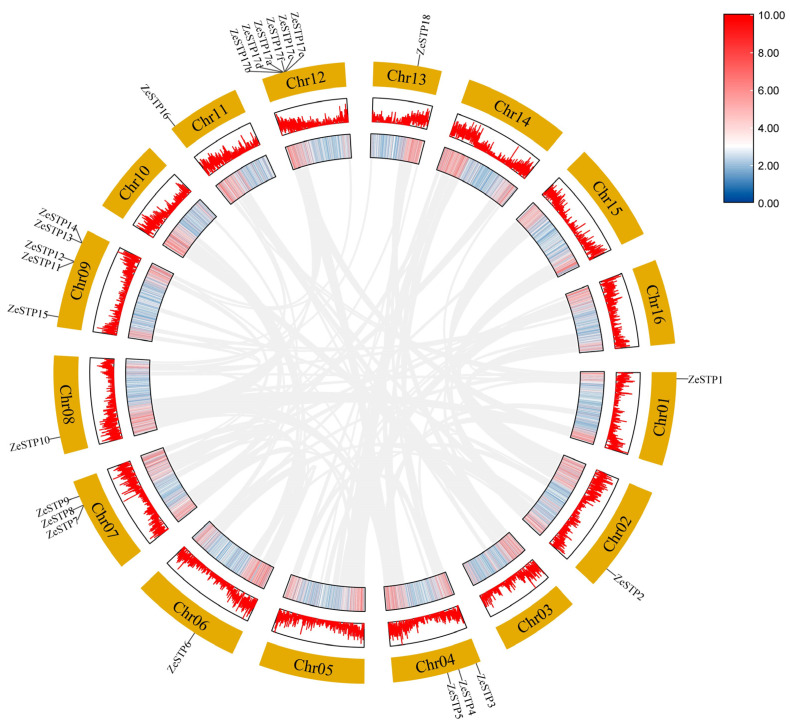
Homology analysis of *ZeSTP* genes in *Z. elliottiana* (chromosomes 1–16). Chromosome-scale localization (outer ring); genomic gene density distribution (middle rings); genome-wide synteny blocks (gray connectors).

To investigate the expansion of the *ZeSTP* gene family, the gene duplication events were examined. In *Z. elliottiana*, multiple pairs of tandemly duplicated genes, *ZeSTP7*/*ZeSTP8*, *ZeSTP12*/*ZeSTP13*, *ZeSTP14*/*ZeSTP15*, and *ZeSTP17a*/*b*/*c*/*d*/*e*/*f*, were identified (Figure 4). However, intraspecific collinearity analysis of the *ZeSTP* gene family in *Z. elliottiana* did not reveal any collinear blocks (Figure 5).

**Figure 6:**
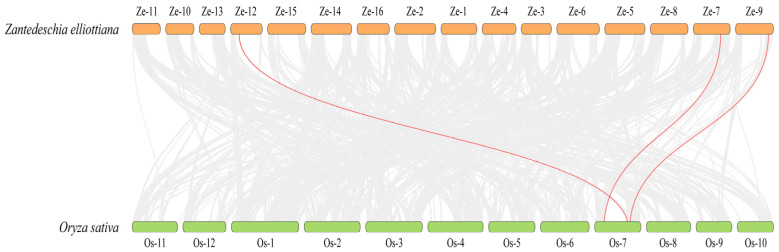
Interspecific collinearity analysis of *STP* genes between *Z. elliottiana* and *O. sativa*. Gray lines indicate collinear blocks between the two species, while red lines represent collinear gene pairs between STP genes of *Z. elliottiana* and *O. sativa*.

Nevertheless, the collinearity analysis between *O. sativa* and *Z. elliottiana*, which are both monocotyledonous plants, was further conducted. The results showed there are three pairs of homologous genes between the two species (Figure 6). Specifically, *ZeSTP8* on chromosome 7 of *Z. elliottiana* and *OsSTP22* on chromosome 7 of *O. sativa* form a significant collinear gene pair. Additionally, *ZeSTP14* on chromosome 9 and *ZeSTP17* on chromosome 12 of *Z. elliottiana* are homologous to *OsSTP6* (*OsMST6*) on chromosome 7 of *O. sativa*. This suggests that the ancestral genes of *ZeSTP14* and *ZeSTP17* likely originated from the same gene. Interestingly, all *ZeSTP* genes in *Z. elliottiana* that showed collinearity with *O. sativa* have undergone duplication events, and the homologous rice genes are all located on chromosome 7 (Figure 6).

### 2.6. Expression Analysis of ZeSTP Genes in Z. elliottiana After Inoculation with P. carotovora subsp. carotovora

**Figure 7:**
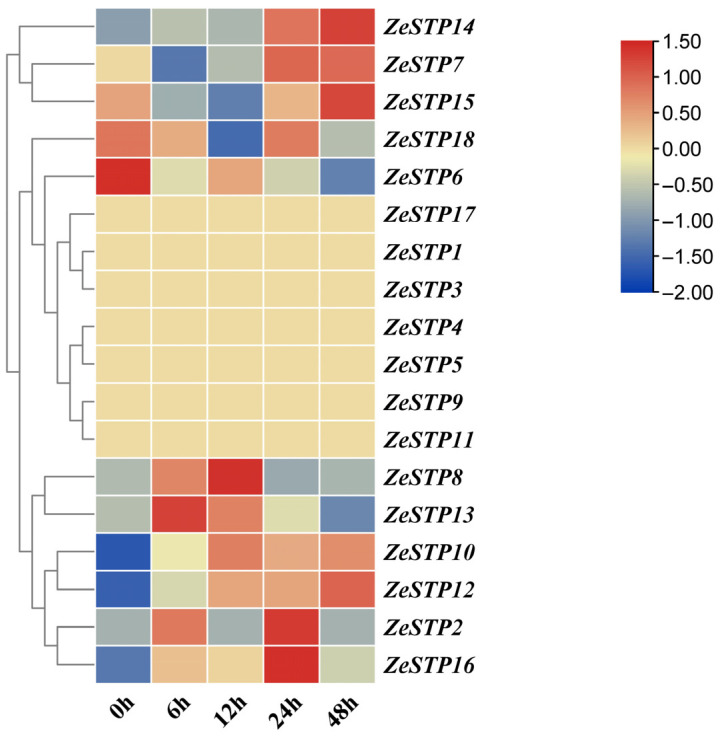
Heatmap of *ZeSTP* gene family expression in the transcriptome data after inoculation with *P. carotovora* subsp. *carotovora*. The gene expression values are log-transformed with base 2. Color marks indicate changes in gene expression: red shows high expression, and blue shows low expression.

In previous studies, the transcriptome data of *Z. elliottiana* cv. Jingcai Yangguang inoculated with *P. carotovora* subsp. *carotovora* was analyzed [32]. To investigate the response of *ZeSTP* genes in *Z. elliottiana* to *P. carotovora* subsp. *carotovora*, the expression levels of *ZeSTP* genes in the transcriptome at 0, 6, 12, 24, and 48 h post-inoculation were analyzed (Figure 7).

The transcriptome heatmap showed that *ZeSTP7*, *ZeSTP14*, and *ZeSTP15* transcript levels increased from 12 to 48 h (Figure 7). *ZeSTP15* exhibited sustained high expression, with 48 h levels 2.76-fold higher than at 12 h. *ZeSTP10* and *ZeSTP12* were also progressively upregulated. These genes, especially *ZeSTP7* and *ZeSTP15*, are likely associated with calla lily soft rot disease infection.

**Figure 8:**
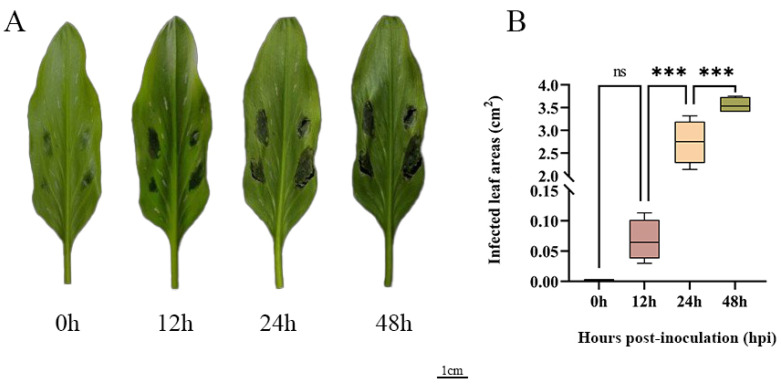
(**A**) Disease phenotypes at different time points after inoculation of *P. carotovora* subsp. *carotovora* on the leaves of *Z. elliottiana* cv. Jingcai Yangguang. (**B**) The lesion areas of the leaves after inoculation at different time points (one-way ANOVA: ns: non-significant; *** p < 0.001).

**Figure 9:**
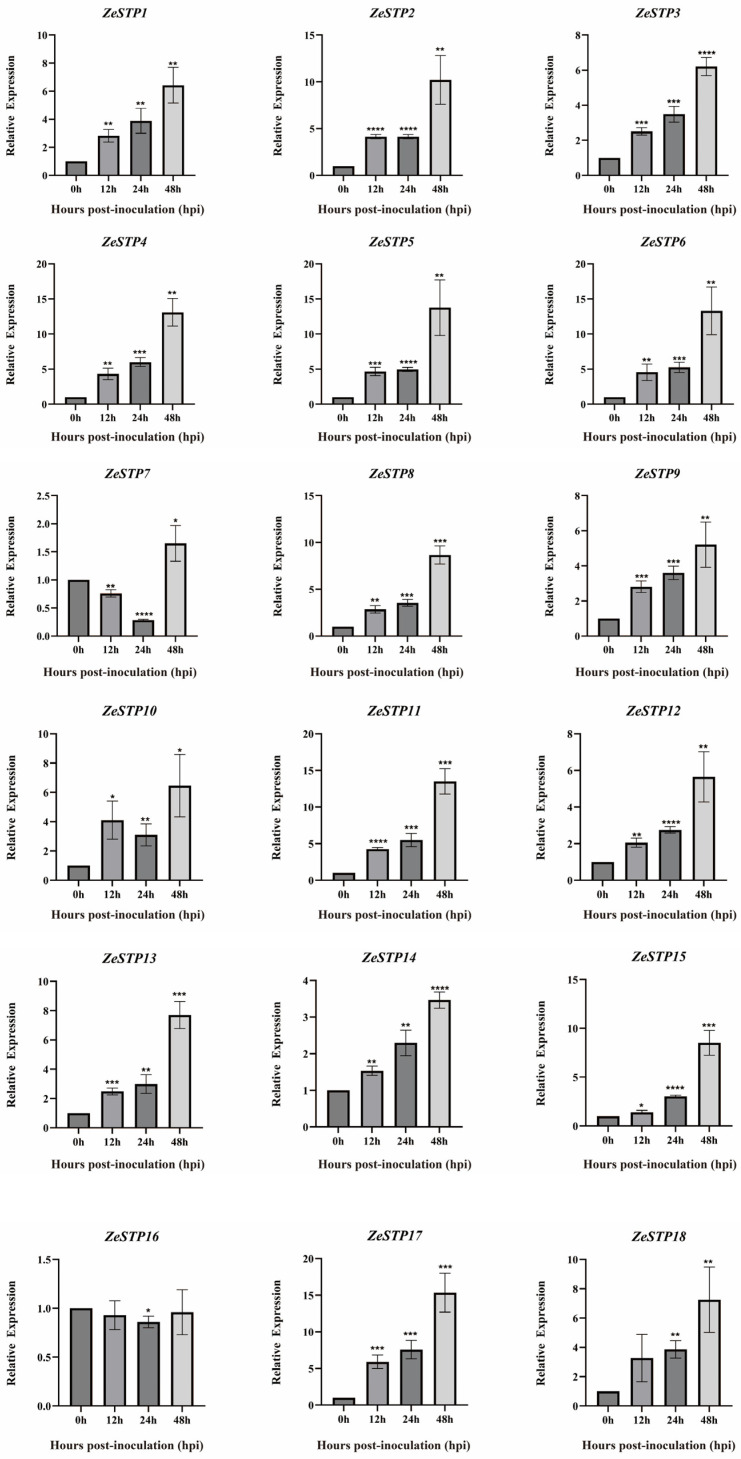
Expression levels of *ZeSTP* family genes in *Z. elliottiana* leaves after *P. carotovora* subsp. *carotovora* inoculation. Expression dynamics (0–48 h post-inoculation) were quantified by RT-qPCR with gene-specific primers. Data represent the mean ± SE (n = 3); asterisks denote significant differences (Student’s *t*-test: * p < 0.05; ** p < 0.01; *** p < 0.001; **** p < 0.0001).

To further explore how *ZeSTP* genes in *Z. elliottiana* respond to *P. carotovora* subsp. *carotovora*, colored calla lily leaves were inoculated with *P. carotovora* subsp. *carotovora* (Figure 8A). Following inoculation, both the symptomatic area and the severity of disease intensified progressively over time (Figure 8B). Symptomatic leaf tissue was sampled as 2 cm diameter discs centered on the inoculation site at 0, 12, 24, and 48 h post-inoculation, and the expression levels of *ZeSTP* genes were measured (Figure 9). Gene expression levels of *ZeSTP15* and *ZeSTP17* rise significantly over time, with *ZeSTP17* expression increasing 5.91-, 7.57-, and 15.35-fold at 12, 24, and 48 h, respectively, compared to 0 h. Conversely, *ZeSTP7* and *ZeSTP16* expression levels dropped 3.57- and 1.17-fold at 24 h but rebounded by 48 h (Figure 9). These results suggest that the *ZeSTP* gene family is involved in the interaction with *P. carotovora* subsp. *carotovora*, with *ZeSTP15* and *ZeSTP17* showing robust responses.

### 2.7. Sugar Transport Properties of ZeSTP7, ZeSTP15, and ZeSTP17

**Figure 10:**
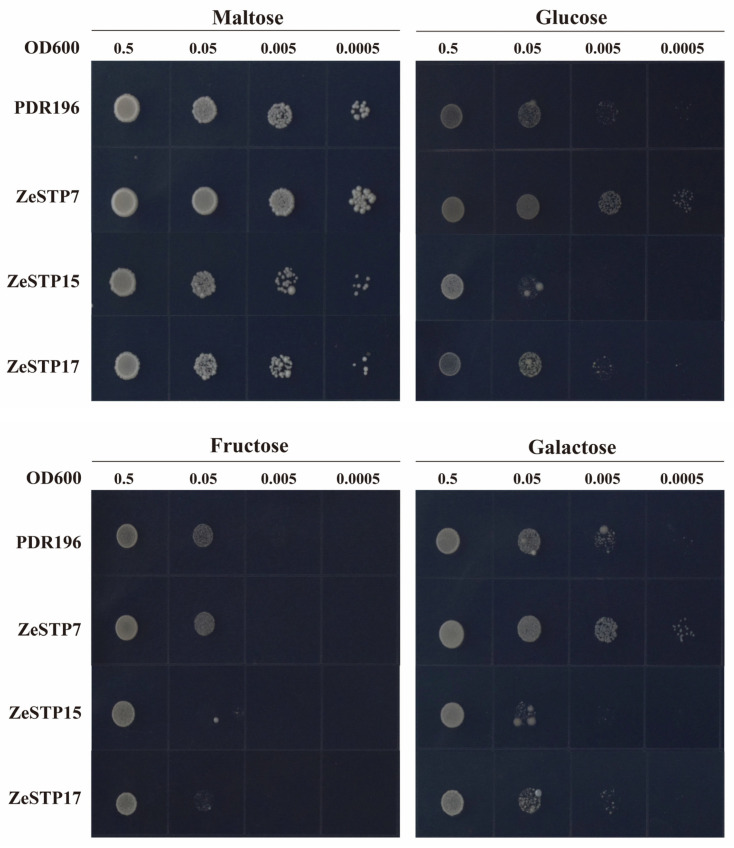
Functional complementation assay of ZeSTP in hexose transporter-null yeast EBY.VW.4000. Transformants expressing PDR196 (empty vector), ZeSTP7, ZeSTP15, or ZeSTP17 were cultured on SD/-Ura medium with 2% maltose, glucose, fructose, or galactose at 30 °C for 72 h.

The hexose transport-deficient yeast strain EBY.VW4000, which lacks over 20 endogenous hexose transporter genes and cannot grow on SD/-Ura medium with hexoses as the carbon source was used to assess the sugar transport activity of ZeSTP proteins in calla lily. Based on genomic and transcriptomic analyses and tandem repeat detection, ZeSTP7, ZeSTP15, and ZeSTP17 were selected for functional characterization. All three proteins enabled growth on SD/-Ura medium with 2% maltose. ZeSTP7 restored growth on media containing glucose and galactose (Figure 10), indicating its capacity to transport these sugars. In contrast, ZeSTP15 and ZeSTP17 showed no growth on media with 2% fructose and only faint growth on media with 2% glucose and 2% galactose, suggesting limited transport ability for these sugars.

## 3. Discussion

The *ZeSTP* gene family is divided into five subgroups, with group III comprising only monocots. This implies that group III members were retained in monocots but lost in dicots following whole-genome duplication. In the collinearity analysis between *O. sativa* and *Z. elliottiana*, *ZeSTP8* and *OsSTP22* were identified as a significant collinear gene pair, while *OsSTP6* (*OsMST6*) is homologous to *ZeSTP14* and *ZeSTP17*. Significant collinearity indicates conserved chromosomal positions and order across species, suggesting a common ancestral origin [33,34,35]. Thus, *ZeSTP8* and *OsSTP22*, as well as *ZeSTP14* and *ZeSTP17* with *OsSTP6*, share evolutionary relatedness. Differential selective pressures have led to motif variations among *ZeSTP* subgroups, while conserved domains remain stable. This suggests that despite sequence divergence, the three-dimensional structures and functional domains of ZeSTP proteins are likely conserved.

The *ZeSTP* gene family is unevenly distributed across the 16 chromosomes of *Z. elliottiana*, likely reflecting gene function, genomic evolution, and intergenic interactions. Tandem duplication events have produced gene pairs such as *ZeSTP7*/*ZeSTP8*, *ZeSTP14*/*ZeSTP15*, and *ZeSTP17a-f*, with *ZeSTP17* retaining six tandem paralogs harboring conserved coding regions. Similar extensive tandem duplications in the *ZePER* gene family were reported by Wang, significantly increasing *ZePER* gene numbers [36]. These tandem repeats are evolutionarily stable, maintaining gene expression patterns and functions [37,38,39]. The multiple tandem repeats likely explain the high expression of *ZeSTP7*, *ZeSTP15*, and *ZeSTP17* in *Z. elliottiana* leaves inoculated with *P. carotovora* subsp. *carotovora*, especially for *ZeSTP17*, suggesting their potential roles in responding to *P. carotovora* subsp. *carotovora*. Wang identified two whole-genome duplication events in *Z. elliottiana*, with paralogous gene pairs of *PER* genes on chromosomes Chr1, Chr2, Chr4, Chr5, Chr8, Chr12, Chr15, and Chr16 [36]. However, intraspecific collinearity analysis revealed no collinear blocks for *ZeSTPs*. Therefore, it is speculated that the *ZeSTP* gene family has undergone purifying selection during evolution, as evidenced by its remarkably conserved sequence architecture, suggesting their critical roles in maintaining essential biological processes within the species.

Sugar transporters in plants are primarily divided into two major categories: SWEETs and STPs. SWEETs mediate photoassimilate partitioning, seed development, and floral nectar secretion. STP proteins mediate monosaccharide transport and are key in intracellular sugar allocation and utilization. These plasma membrane H+/monosaccharide symporters transport a broad range of substrates, including glucose, galactose, fructose, and pentoses [40].

*AtSTP13* is crucial for *A. thaliana* disease resistance, with expression upregulated upon infection by necrotrophic pathogens like *B. cinerea* and *P. syringae*. Overexpression of *AtSTP13* can enhance the plant’s ability to uptake glucose from the apoplast and increase resistance to *B. cinerea* [12,22,41]. Similarly, VvHT5, an AtSTP13 ortholog, restricts fungal sugar utilization by promoting extracellular monosaccharide reabsorption, enhancing grapevine resistance to gray mold [42]. In this study, hexose mutant yeast assays showed that ZeSTP7 transports glucose and galactose, while ZeSTP15 and ZeSTP17 exhibit limited capacity for these sugars. Interestingly, ZeSTP7 clusters with AtSTP13 in the phylogenetic tree, and it is highly expressed following inoculation with *P. carotovora* subsp. *carotovora*, suggesting it participates in colored calla lily–*P. carotovora* subsp. *carotovora* interactions by transporting glucose and galactose. Conversely, TaSTP13, an AtSTP13 ortholog, increases wheat susceptibility to stripe rust and powdery mildew by elevating cytoplasmic hexose levels [25]. The glucose-transport-deficient mutant HvSTP13GR enhances barley resistance to leaf rust and powdery mildew [43]. Thus, STPs can enhance plant resistance by promoting defense responses or depriving pathogens of sugars, or increase susceptibility by facilitating pathogen sugar acquisition [44,45].

During plant–pathogen interactions, *STP* genes are activated by defense signaling components, hormonal pathways, and metabolic regulators. *OsMST6* expression, upregulated by salt stress and sugar, is high during early and mid-grain formation in rice and declines thereafter [46]. Activated by the transcription factor OsERF120, *OsMST6* enhances cold tolerance in rice seedlings by regulating sugar and ABA signaling pathways during cold stress [47]. Collinearity analysis between *Z. elliottiana* and *O. sativa* identified *OsSTP6* (*OsMST6*) as homologous to *ZeSTP14* and *ZeSTP17*. Collinear genes often share biological functions and participate in common metabolic or signaling pathways. In this study, *ZeSTP17* and *ZeSTP15* expression levels significantly increased following inoculation, suggesting their functional analogy to *OsMST6*, potentially mediating sugar transport during host–pathogen interactions through transcriptional or signaling regulation, while possibly responding to abiotic stresses.

The defense responses of plants to *P. carotovora* subsp. *carotovora* are associated with changes in gene expression and the metabolome level. These changes also can be consider as specific responses of plants to biotic stress [48]. Transcriptome analysis of *Pinellia ternata* shows that 20 h after infection by *P. carotovora*, the expression levels of genes encoding cell wall proteins and enzymes and proteins involved in defense mechanisms during biotic stress increase, while the expression levels of genes for pectin lyase, expansin, and *PR1* decrease [49]. When tomato plants are infected with *P. carotovora* subsp. *carotovora* for 24 h, the expression levels of *MYB*, *EREBP*, *PR3*, and *PR6* genes significantly increase [50]. Similarly, 24 h after inoculation of pepper plants with *P. carotovora* subsp. *carotovora*, 13 key induced defense genes (such as *PR1* and *PAL*) are significantly upregulated [51]. In the transcriptome analysis of this study, some *ZeSTPs* genes, such as *ZeSTP6* and *ZeSTP18*, responded during the 0–6 h post-inoculation period. During the 12–48 h post-inoculation period, the primary responses may trigger a series of secondary reactions, leading to the upregulation of *ZeSTP7*, *ZeSTP15*, and *ZeSTP17*. Alternatively, changes in *ZeSTPs* may induce alterations in other pathways or genes, and in the later stages of infection, *ZeSTPs* may act in synergy or antagonism with other pathways.

Previous studies have shown that *P*. *carotovorum* invasion activates jasmonic acid (JA), ethylene (ET), and salicylic acid (SA) signaling pathways. In transgenic *Arabidopsis* overexpressing the bacterial *expI* gene, SA-dependent defense genes *PR1*, *NPR1*, and *NPR4* were significantly induced [52]. In Chinese cabbage, JA/ET-responsive transcription factors of the ERF and WRKY families, including *WRKY33*, were upregulated upon *P. carotovora* infection [53], suggesting these pathways are likely conserved in our system. Additionally, *STPs* may cooperate with defense genes post pathogen challenge: *PDF1.2* and *PAD3* were strongly induced within 48 h after *Botrytis cinerea* inoculation, with concurrent *AtSTP13* expression [12]. The wild-type *MtSTP13.1* restricts apoplastic hexose availability, thereby activating pathogenesis-related (*PR*) and flavonoid biosynthesis genes to enhance basal resistance against *Erysiphe pisi* in pea [54]. Here, it shows that *ZeSTPs* likely participate in *Z. ellioltiana–P. carotovora* subsp. *carotovora* interactions. This study is not an exhaustive investigation of the defense mechanisms of *Z. ellioltiana* against *P. carotovora* subsp. *carotovora* but rather a preliminary assessment of the response of *ZeSTPs* to *P. carotovora* subsp. *carotovora*. The mechanisms involved will be addressed in subsequent studies.

In this study, although our gene-expression profiling uncovered the overall regulatory trend of the *ZeSTP* family during soft rot infection, the pooled samples unavoidably contained both pathogen-attacked cells and adjacent, non-infected cells, as well as different tissue layers (epidermis, cortex, vasculature). Such bulk analysis masks cell-type-specific or local expression patterns and ignores the spatiotemporal dynamics of these genes. To fully elucidate the function of the *ZeSTP* family, future work should apply single-cell RNA-seq and/or in situ hybridization to resolve expression at the cellular scale.

## 4. Materials and Methods

### 4.1. Plant Materials and Treatments

The experimental material used was *Z*. *elliottiana* cultivar Jingcai Yangguang, grown in the greenhouse of the Beijing Academy of Agriculture and Forestry Sciences, with ambient conditions of 25 °C, 60% relative humidity, and a photoperiod of 16 h light/8 h dark.

Fully expanded leaves were cut from the base of the petiole and soaked in 0.5% sodium hypochlorite for 20 min, then rinsed with sterile distilled water and air-dried. The inoculation of *P. carotovora* subsp. *carotovora* was performed according to the method described by Luzzatto [55]. The soft rot bacteria were streaked on LB agar and cultured at 28 °C for 16 h. A single colony was selected and cultured in 4 mL of LB liquid medium at 28 °C with shaking at 150 rpm for 10 h. The bacterial culture (2 mL) was centrifuged at room temperature at 12,000 rpm for 3 min. The supernatant was discarded, and 1 mL of fresh LB liquid medium was added (C1). After vortexing, 0.1 mL was transferred to 0.9 mL of fresh LB liquid medium (C2) and vortexed again. The OD_600_ value was measured using a spectrophotometer. Based on the formula OD_600_ = 1.0 (10^9^ CFU) [56], the concentration of C1 was calculated. C1 was diluted to a working concentration of 10^7^ CFU/mL with double-distilled water for injection. The bacterial suspension (0.1 mL) was inoculated at four symmetrical points along the midrib on the underside of the leaf. The inoculated plant material was cultured at 28 °C, and symptoms were recorded at 0, 12, 24, and 48 h. Leaf discs (2 cm in diameter, centered on the inoculation site) were harvested; all samples were frozen in liquid nitrogen and stored at −80 °C. Lesion areas post-inoculation were quantified using ImageJ 1.54 software https://imagej.nih.gov/ij/ (accessed on 30 June 2025).

### 4.2. Identification and Physicochemical Property Analysis of Sugar Transporter Protein-Encoding Genes in Calla Lily

Based on the annotation file of the genome data of *Z. elliottiana* cv. Jingcai Yangguang [32], the protein sequences of 45 AtSTPs were used as references for BLASTp https://blast.ncbi.nlm.nih.gov/Blast.cgi (accessed on 20 May 2025) analysis [57]. These sequences were compared with the entire proteome of the colored calla lily to identify potential *ZeSTP* genes. The bioinformatics tool ExPASy-ProtParam http://web.expasy.org/protparam/ (accessed on 20 May 2025) was employed to analyze the coding sequence length, amino acid sequence length, molecular weight, and isoelectric point of the STP gene family in colored calla lily. The transmembrane domains of ZeSTP proteins were predicted using the online tool DeepTMHMM https://dtu.biolib.com/DeepTMHMM (accessed on 25 June 2025).

### 4.3. Construction of the Phylogenetic Tree and Structural Analysis of the ZeSTP Gene Family

The complete amino acid sequences of rice and *A. thaliana* were downloaded from the rice genome database http://plants.ensembl.org/Oryza_sativa/Info/Index (accessed on 24 June 2025) and *A. thaliana* database https://www.arabidopsis.org/ (accessed on 20 May 2025), respectively. The protein sequences of OsSTPs and AtSTPs were compared with those of ZeSTPs. A multiple sequence alignment of STP proteins was performed using MUSCLE, and a phylogenetic tree was constructed using the neighbor-joining method in MEGA 11 with 1000 bootstrap replicates. The tree was beautified using the online tool Evolview v4 https://evolgenius.info/evolview/#/treeview (accessed on 25 June 2025).

Based on the annotation file of the Jingcai Yangguang genome data, the gene annotation files of *ZeSTPs* were obtained. The gene structure patterns were visualized using TBtools-II v2.310 software [58] and combined with the phylogenetic tree of the *ZeSTP* gene family for presentation.

### 4.4. Conserved Motif and Domain Analysis of ZeSTP Proteins

The MEME Suite version 5.5.7 https://meme-suite.org/meme/ (accessed on 25 June 2025) was used to search for conserved motifs, with the maximum number of motifs set to 20. Based on the results generated by MEME, the conserved motif models were redrawn using TBtools-II. The conserved domain results of ZeSTP proteins were obtained through NCBI https://www.ncbi.nlm.nih.gov/Structure/bwrpsb/bwrpsb.cgi (accessed on 20 May 2025), visualized using TBtools-II, and combined with the phylogenetic tree of the *ZeSTP* gene family for presentation.

### 4.5. Chromosomal Localization Analysis of the ZeSTP Gene Family

The chromosomal positions of *ZeSTPs* were obtained based on the chromosome annotation file and visualized using TBtools-II software. The *ZeSTP* gene family was named according to the order of their appearance on the chromosomes. The tandem gene pairs within the *ZeSTP* gene family were identified using the One Step MCScanX module in TBtools-II, with CPU for BlastP set to 2, E-value set to 1 × 10^−3^, and Number of Blast hits set to 10.

### 4.6. Intraspecific and Interspecific Collinearity Analysis

The whole-genome data file and gene annotation file of colored calla lily were obtained. The whole-genome data file and gene annotation file of rice were retrieved from the rice genome database (https://plants.ensembl.org/Oryza_sativa/Info/Index) (accessed on 24 June 2025). One Step MCScanX in TBtools-II was used for intraspecific collinearity analysis of colored calla lily and interspecific collinearity analysis between colored calla lily and rice, with CPU for BlastP set to 2, E-value set to 1 × 10^−3^, and Number of Blast hits set to 10.

### 4.7. Quantitative Analysis

Total RNA was extracted from the leaves of colored calla lily at 0 h, 12 h, 24 h, and 48 h post-inoculation using the EASYspin Plus Plant RNA Kit (Aidlab, Beijing, China). cDNA was synthesized according to the manufacturer’s instructions using the HiScript II First Strand cDNA Synthesis Kit (Vazyme, Nanjing, China). RT-qPCR was performed using the ChamQ SYBR qPCR Master Mix (Vazyme, China). The expression levels of *ZeSTP* genes were calculated using the 2^−ΔΔCT^ method [59]. *ZeActin* was used as a reference gene to normalize the expression levels of *ZeSTP* genes. Primers were designed using Primer Premier 5 (Table A1).

### 4.8. Expression Analysis of ZeSTP Genes in Colored Calla Lily After P. carotovora subsp. carotovora Inoculation

Based on the transcriptome data of colored calla lily ‘Jingcai Yangguang’ [32], the expression heatmap of *ZeSTP* genes in the transcriptome after inoculation was created using TBtools-II software. The expression levels of *ZeSTP* genes after *P. carotovora* subsp. *carotovora* inoculation were measured by RT-qPCR, with three independent replicates for each gene at each time point. *ZeSTP* genes from 0 to 48 h post-inoculation were plotted using GraphPad Prism 9.5, and significant differences were marked at * *p* < 0.05; ** *p* < 0.01; *** *p* < 0.001; **** *p* < 0.0001 using independent *t*-tests.

### 4.9. Sugar Transport Activity of ZeSTP7, ZeSTP14, and ZeSTP17 in Hexose-Mutant Yeast

To investigate the heterologous expression of ZeSTPs in yeast, pDR196, pDR196-*ZeSTP7*, pDR196-*ZeSTP15*, and pDR196-*ZeSTP17* were transformed into the hexose-mutant yeast strain EBY.VW.4000, respectively [60]. The transformed yeast cultures were adjusted to an OD_600_ of 0.5 and diluted 10, 100, and 1000 times. Then, the yeast cultures were spotted on SD/-Ura media containing 2% maltose, 2% glucose, 2% fructose, and 2% galactose, respectively. The media were incubated at 30 °C for 3 days, and the growth of yeast was observed.

## 5. Conclusions

In summary, 18 *ZeSTP* genes were identified from colored calla lily and divided into five subgroups, all predicted to encode membrane-localized transporters. Genomic analyses revealed nine tandem duplication events within the *ZeSTP* family and three collinear orthologs with *OsSTPs*. Temporal expression profiling under the *P. carotovora* subsp. *carotovora* inoculation demonstrated significant upregulation of *ZeSTP7*, *ZeSTP15*, and *ZeSTP17*. Functional complementation in hexose transporter-deficient yeast confirmed ZeSTP7-mediated glucose/galactose transport, while ZeSTP15/17 exhibited limited substrate specificity. Integrated characterization—including physicochemical profiling, phylogenetic reconstruction, and synteny analysis—provides evolutionary insights into *ZeSTP* family expansion. These findings establish a foundation for functional dissection of *ZeSTP* genes in colored calla lily–pathogen interactions.

## Figures and Tables

**Table 1:** Physicochemical properties of STP gene family members in *Z. elliottiana* ^1^.

Gene Name	Gene ID	CDS (bp)	Amino Acid	MW (kDa)	pI	TMD
ZeSTP1	Zh01G023300	396	131	15.1	10.86	2
ZeSTP2	Zh02G215400	330	109	12	5.5	2
ZeSTP3	Zh04G018400	1539	512	55.9	8.96	12
ZeSTP4	Zh04G074400	318	105	11.5	5.1	2
ZeSTP5	Zh04G094400	591	196	21	10.37	2
ZeSTP6	Zh06G150400	258	85	9.1	8.71	2
ZeSTP7	Zh07G168800	1599	532	58.1	9.35	12
ZeSTP8	Zh07G169400	1527	508	55.9	6.34	12
ZeSTP9	Zh07G201000	1533	510	55.5	8.98	12
ZeSTP10	Zh08G065200	1560	519	56.3	9.03	12
ZeSTP11	Zh09G034900	399	132	14.1	9.69	1
ZeSTP12	Zh09G113600	1566	521	56.1	9.21	12
ZeSTP13	Zh09G113700	2463	820	91.9	9.24	8
ZeSTP14	Zh09G158700	1554	517	56.6	9.4	12
ZeSTP15	Zh09G158800	1554	517	56.5	9.05	12
ZeSTP16	Zh11G017400	1572	523	54.7	9.78	12
ZeSTP17	Zh12G088700	1557	518	56.5	8.96	12
ZeSTP18	Zh13G066500	1185	394	42.2	9.74	9

^1^ CDS, coding sequence; MV, viscosity average molecular weight.

## Data Availability

Data are contained within the article.

## Appendix A

**Table A1:** The primer sequences for RT-qPCR used in this study.

Primer	Forward (5′ to 3′)	Reverse (5′ to 3′)
*ZeSTP1*-qRT	TGCCACCTCTGCCATCAT	GCCAGTTGGGCTTGCTAT
*ZeSTP2*-qRT	CTGCTTCCTCGCCTACCA	GCGTCCCACCTGTTGTTA
*ZeSTP3*-qRT	CGTTGGCATCGGTTTCGC	TCCGCCCAGGATCTTGTT
*ZeSTP4*-qRT	GGGGAGGTCAGGGAAGTA	CAGTTGGAAGCCGATGTT
*ZeSTP5*-qRT	CTAGTGCTATGTGCGCTCTTT	GCTCTTGCTCTGACCCTGT
*ZeSTP6*-qRT	GGGGAGGTCGGGAAAGTA	AAGATGGAGCCAAGGATGA
*ZeSTP7*-qRT	CATCGCACAAGCCTTCCTC	GATGGGCACGTTCTTGGTCT
*ZeSTP8*-qRT	GGTCTTCCAGCAGTTCACG	GACGCAGGCTTCTAGCAAT
*ZeSTP9*-qRT	GCTTCGCAAACCAGTCGG	TCTTGTTGGCAGCGTAGTTCA
*ZeSTP10*-qRT	GAGATGGCAAGGCAGGTCC	CACGGGCGAGTAGAACAGG
*ZeSTP11*-qRT	GCATAGTAGCGTCCAGTCG	CTTGGCGGTGTTGTAGTTC
*ZeSTP12*-qRT	TAACGTCTTCGCCACCTTCG	ATTTGCCCTCGCCGCTCA
*ZeSTP13*-qRT	TGGAGCCCGCTGAAAGGT	TGGCGTGTCAATGTATCG
*ZeSTP14*-qRT	CCAACATCCTGAAGCGAAAG	CGGTGATAACGGCGGACA
*ZeSTP15*-qRT	CGACATCCACGCCGAGTT	GGAAGAAGGGAATGAGCACC
*ZeSTP16*-qRT	ACTGGCGGTGCTACTGCTGA	AGGAACACGCCGTACTTGAGG
*ZeSTP17*-qRT	CATCGGGCGTATCCTTCTG	ATCTTGTTGGCTGCGTAGTTC
*ZeSTP18*-qRT	GGCTACGACATCGGCATTT	TCTTGGACTGGGAGTTCTTCTT
*ZeActin*-qRT	ATTTATGAGGGTTATGCTCTTCC	GGAGGAACTGCTCTTGGCTGTCT
pDR196	CCCAGTCACGACGTTGTAAAACG	
pDR196-*STP7*	tcccccgggctgcaggaattcATGCCGGCCGGCGGGTTC	ggtaccgggccccccctcgagGTTGGCACCGTTGTGGCC
pDR196-*STP15*	tcccccgggctgcaggaattcATGGCGGGCGGGGCGTTC	ggtaccgggccccccctcgagGACCACATAGGCGGTCTTCTTG
pDR196-*STP17*	tcccccgggctgcaggaattcATGGCCGGGGGAGCCTTC	ggtaccgggccccccctcgagTACATGGCCGTGGGCCCC
